# Fast Low-Sidelobe Pattern Synthesis Using the Symmetry of Array Geometry

**DOI:** 10.3390/s24134059

**Published:** 2024-06-21

**Authors:** Ming Zhang, Yongxi Liu, Haidong Zhou, Anxue Zhang

**Affiliations:** 1 School of Information and Communications Engineering, Xi’an Jiaotong University, Xi’an 710049, China; liuyongxi@stu.xjtu.edu.cn (Y.L.); anxuezhang@xjtu.edu.cn (A.Z.); 2 Leihua Avionics Institute of AVIC, Wuxi 214062, China; zhouhd@avic.com

**Keywords:** array pattern synthesis, amplitude weighting, symmetry of array geometry, dynamic range ratio, second-order cone programming (SOCP)

## Abstract

Array pattern synthesis with low sidelobe levels is widely used in practice. An effective way to incorporate sensor patterns in the design procedure is to use numerical optimization methods. However, the dimension of the optimization variables is very high for large-scale arrays, leading to high computational complexity. Fortunately, sensor arrays used in practice usually have symmetric structures that can be utilized to accelerate the optimization algorithms. This paper studies a fast pattern synthesis method by using the symmetry of array geometry. In this method, the problem of amplitude weighting is formulated as a second-order cone programming (SOCP) problem, in which the dynamic range of the weighting coefficients can also be taken into account. Then, by utilizing the symmetric property of array geometry, the dimension of the optimization problem as well as the number of constraints can be reduced significantly. As a consequence, the computational efficiency is greatly improved. Numerical experiments show that, for a uniform rectangular array (URA) with 1024 sensors, the computational efficiency is improved by a factor of 158, while for a uniform hexagonal array (UHA) with 1261 sensors, the improvement factor is 284.

## 1. Introduction

Sensor arrays play a key role in modern information processing systems due to their powerful spatial filtering ability. They have been successfully applied to various fields such as radar [1], wireless communication [2], satellite navigation [3], radio astronomy [4], speech enhancement [5], and seismic exploration [6]. Array patterns with low sidelobes are very important in these applications to cope with unwanted interferences [7,8,9]. One of the most widely used methods to synthesize patterns with low sidelobes is amplitude weighting (also called amplitude taper). Conventional analytical methods such as Dolph–Chebychev weighting [10,11] and Taylor weighting [12,13] can be implemented very efficiently. However, these methods are limited to arrays with specific geometries and cannot take into account the sensor patterns. Numerical optimization-based techniques, which address the above issues, have attracted extensive attention in amplitude weighting. Apart from the ability to handle arrays with arbitrary geometries and sensor patterns, they can deal with additional constraints, such as forming nulls at some specified directions or controlling the dynamic range of the weighting coefficients. While heuristic algorithms [14,15] exhibit robust modeling and generalization capabilities, they are relatively time-consuming and cannot guarantee that optimal results will be obtained. Convex optimization shows supreme performance in finding the optimal solutions at the expense of modeling difficulty. Due to the quadratic nature of the array pattern expression, second-order cone programming (SOCP) and semidefinite programming (SDP) have gained widespread applications in array pattern synthesis [16,17,18,19,20,21,22,23,24,25]. Nevertheless, for convex optimization methods, the problem models vary with the optimization purposes, and most problems appear to be non-convex. Relaxation must be adopted to transform the original problems into their convex approximations. For example, in [17], the minimization of the $l_{1}$-norm, instead of the non-convex $l_{0}$-norm, is adopted to design sparse arrays via sequential convex optimizations. In [19], cross-polarization is considered in power pattern synthesis using semidefinite relaxation. In [24], phase-only beamforming with continuous or discrete shifters is also solved by semidefinite relaxation. However, the computational cost of these methods is very high, particularly for large-scale arrays used in modern radar and communication systems [26,27,28]. Consequently, there is an urgent need to reduce their computational burden.

Although many modeling methods have been proposed for array pattern synthesis using convex optimization, little attention has been paid to their efficient implementations. For example, most optimization problems are solved by available packages such as CVX [29] or SeDumi [30]. In some cases, however, these packages are not effective enough, since redundant variables will be introduced when transforming a given problem into a standard one. In addition, the computational complexities of SOCP and SDP are $N^{3}$ and $N^{3.5}$ per iteration, respectively, with *N* being the dimension of the problem [31,32], while for most problems, 20∼40 iterations are required [33]. When semidefinite relaxation is employed [34], the dimension of the problem is increased from *N* to $N^{2}$, leading to very high computational complexity. As a result, for large-scale arrays with thousands of sensors and tens of thousands of constraints that are commonly encountered in military applications, the SOCP solvers will take several hours to find the solution on a personal computer, while the SDP solvers do not work. Therefore, the computational efficiency is very important for these problems.

Fortunately, sensor arrays as well as their patterns used in practice usually have symmetric structures [35]. For example, there is mirror symmetry in uniform linear arrays (ULAs) and uniform rectangular arrays (URAs), and rotational symmetry in uniform hexagonal arrays (UHAs) and concentric circular arrays (CCAs). Moreover, to simplify the feeding network, large-scale arrays are usually divided into several identical blocks [36]. Thus, the weighting coefficients also have symmetric distributions. Utilizing these symmetries, both the dimension of the optimization variables and the number of constraints in the optimization problems can be reduced. As a consequence, the computational efficiency can be improved dramatically. The conjugate weighting for ULAs with central symmetry is analyzed in [36,37]. However, a detailed discussion on the mirror symmetry and rotational symmetry is not presented. In [38], only the central symmetry is used to optimize a URA. Therefore, the mirror symmetry of the array and its beampattern is not fully exploited. In [39], a thinned array is optimized and the results show that the excitation coefficients exhibit rotational symmetry. Nevertheless, this symmetry is not considered in the design procedure and thereby the computational efficiency can be improved.

This paper studies a fast amplitude weighting method based on the SOCP by leveraging these symmetries. We mainly focus on the planar arrays with mirror symmetry (e.g., URAs) and rotational symmetry (e.g., UHsA). A URA located on the xy-plane is symmetric about the *x*-axis as well as the *y*-axis. Therefore, only the weighting coefficients located in the first quadrant need to be optimized. A UHA located on the xy-plane is symmetric about the *x*-axis as well as the lines passing through the origin with angles of $\phi =60^{\circ }$ and $\phi =120^{\circ }$. Therefore, only the weighting coefficients located in the region of $0^{\circ }\leq \phi <60^{\circ }$ need to be optimized. Moreover, due to the symmetry of array pattern with respect to the azimuth angle, the number of constraints can also be reduced. Based on these considerations, the dimension of the optimization variables can be reduced by 75% for URAs and by 83% for UHAs. In addition, to control the dynamic range of array excitations that can simplify the feeding network and improve the robustness of the array when performing beam scanning [40,41,42], the lower and upper bounds of the weighting coefficients are taken into account in the proposed method.

In order to verify the efficiency of the proposed method, we implemented the algorithm described in [31,43] for SOCP problems without performing any code optimization. Two URAs with 256 sensors and 1024 sensors and two UHAs with 331 sensors and 1261 sensors are tested in the simulation experiments. The results show that the computational efficiency is improved by an order of magnitude for URA-256 and UHA-331, and by two orders of magnitude for URA-1024 and UHA-1261. Moreover, it is found that the direct implementation of the SOCP solver described in [31,43] is more efficient than the CVX package when solving the problems of amplitude tapering.

The main contributions of this paper are as follows:

1. We propose a fast low-sidelobe pattern synthesis method using the symmetry of array geometry, which reduces the dimension of the optimization variables and improves the computational efficiency significantly.
2. The lower and upper bounds of the weighting coefficients can also be specified in the proposed method, which can control the dynamic range of the weighting coefficients.

The remainder of this paper is organized as follows. Section 2 formulates the amplitude weighting with dynamic range constraints as an SOCP problem. Section 3 describes the method that utilizes the symmetry of URAs and UHAs to reduce the dimension of the optimization problem obtained in Section 2. Simulation experiments are given in Section 4 to validate the effectiveness of the proposed method, followed by a conclusion and discussion in Section 5.

Notations: We use lowercase letters (e.g., *a*), lowercase boldface letters (e.g., a), and uppercase boldface letters (e.g., A) to represent scalars, vectors, and matrices, respectively. The superscripts ${\left(\right)}^{T}$ and ${\left(\right)}^{H}$ denote the transpose and Hermitian transpose, respectively. The real part and imaginary part of a complex number *z* are denoted by Re(z) and Im(z), while $æ=\sqrt{-1}$ is the imaginary unit.

## 2. Problem Formulation

**Figure 1:**
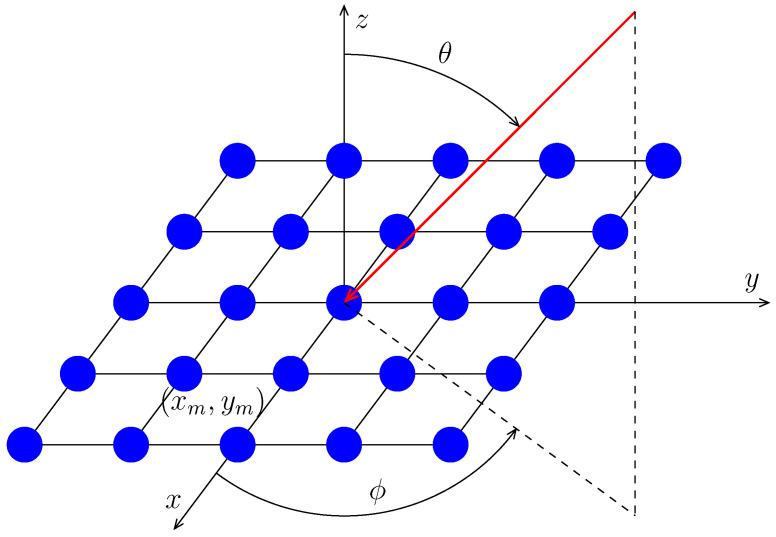
A planar array located on the xy-plane.

Consider a planar array located on the xy-plane as shown in Figure 1. The problem of amplitude weighting that minimizes the peak sidelobe level (PSLL) of the array pattern can be formulated as

(1a) $$\begin{array}{cc} \min_{w\in R^{M}}\; & \max_{\left(\theta ,\phi \right)\in \Theta_{\mathrm{sl}}}\left|w^{H}v\left(\theta ,\phi \right)\right|, \end{array}$$

(1b) $$\begin{array}{cc} s.t.\; & \left|w^{H}v\left(\theta_{0},\phi_{0}\right)\right|=1, \end{array}$$

where w is the weighting coefficients, *M* is the number of sensors, $\Theta_{\mathrm{sl}}$ is the region of sidelobes, v(θ,ϕ) is the steering vector in direction (θ,ϕ), with $\left(\theta_{0},\phi_{0}\right)$ being the direction of the main beam. For planar arrays whose sensors lie in the xy-plane, the *m*th entry of v(θ,ϕ) is given by [37] (ch. 4)

(2) $$f_{m}\left(\theta ,\phi \right)e^{æ\frac{2\pi }{\lambda }\left[x_{m}\sin\left(\theta \right)\cos\left(\phi \right)+y_{m}\sin\left(\theta \right)\sin\left(\phi \right)\right]},$$

where $f_{m}\left(\theta ,\phi \right)$ and $\left(x_{m},y_{m}\right)$ are the pattern and location of the *m*th sensor, respectively, and λ is the wavelength of the operating frequency.

Because there are infinitely many directions in $\Theta_{\mathrm{sl}}$, one has to approximate $\Theta_{\mathrm{sl}}$ by finite samples. For example, by sampling $\Theta_{\mathrm{sl}}$ with *N* points ${\left\{\left(\theta_{n},\phi_{n}\right)\right\}}_{n=1}^{N}$, Problem (1) can be approximated by [44]

(3a) $$\begin{array}{cc} \min_{g\in R^{+},w\in R^{M}}\; & g, \end{array}$$

(3b) s.t.Rev0Hw=1,

(3c) $$\begin{array}{cc} \; & \;\left|v_{n}^{H}w\right|\leq g,\;n=1,\cdots ,N, \end{array}$$

where $v_{n}=v\left(\theta_{n},\phi_{n}\right)$. Note that the constraint $|v_{0}^{H}w|=1$ has been replaced by Re(v0Hw)=1, which does not change the optimal solution [42].

In addition, to prevent large weighting coefficients that make the synthesized pattern sensitive to array errors [37] (ch. 2), one can impose the range constraints as follows:

(4a) $$\begin{array}{cc} \min_{g\in R^{+},w\in R^{M}}\; & g, \end{array}$$

(4b) s.t.Rev0Hw=1,

(4c) $$\begin{array}{cc} \; & \;\left|v_{n}^{H}w\right|\leq g,\;n=1,\cdots ,N, \end{array}$$

(4d) $$\begin{array}{cc} \; & \alpha_{m}\leq w_{m}\leq \beta_{m},\;m=1,\cdots ,M. \end{array}$$

There are two advantages by imposing the range constraints: reducing the complexity of feeding network and increasing the robustness of beam scanning (e.g., maintaining a low PSLL). The cost is adding more constraints in the optimization problem and thus increasing the computational complexity. Because *M* and *N* are very large for planar arrays (e.g., *M* is on the order of $10^{2}$∼$10^{3}$ and *N* is on the order of $10^{4}$), solving Problem (4) efficiently is very important.

Since the steering vectors $v_{n}$ are complex, we have to convert them into real ones to represent Problem (4) in real variables. For example, let

(5) v^n=Re(vn)Im(vn),v˜n=Im(vn)−Re(vn),w^=Re(w)Im(w).

Then we have [45]

(6) RevnHw=v^nTw^andvnHw=[v^n,v˜n]Tw^.

Because Im(w)=0, Problem (4) is equivalent to

(7a) $$\begin{array}{cc} \min_{g\in R^{+},w\in R^{M}}\; & g, \end{array}$$

(7b) s.t.[Re(v0)]Tw=1,

(7c) [Re(vn),Im(vn)]Tw≤g,

(7d) $$\begin{array}{cc} \; & \alpha_{m}\leq w_{m}\leq \beta_{m}, \end{array}$$

where n=1,⋯,N and m=1,⋯,M. In what follows, we use Problem (7) to illustrate how to utilize the symmetry of array geometry. To help understand the problem model, the meanings of variables and parameters in Problem (7) are summarized in Table 1.

Problem (7) can be translated into an SOCP problem and solved by the primal–dual interior-point methods [31]. Because the SOCP problem is convex, its optimal solution is guaranteed. The dimensions of the primal and dual variables of (7) are $D_{p}=2M+3N+1$ and $D_{d}=M+1$, respectively. Because the main computations of the SOCP solver come from (i) constructing a $D_{d}\times D_{d}$ matrix from a $D_{d}\times D_{p}$ matrix and (ii) solving two linear systems of equations with dimension $D_{d}$, the computational complexity of (7) can be reduced if we can reduce $D_{p}$ and $D_{d}$.

## 3. The Proposed Method

### 3.1. Fast Amplitude Weighting for URA Using Mirror Symmetry

**Figure 2:**
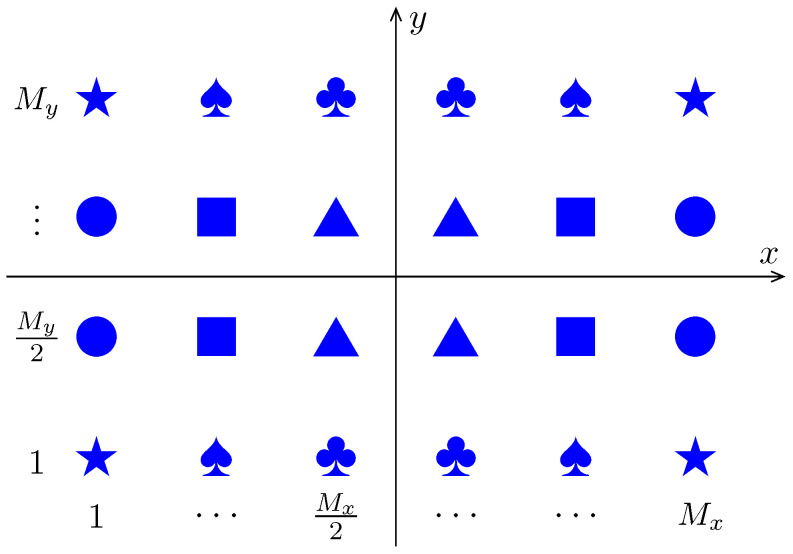
The symmetry of a URA with $M_{x}$ and $M_{y}$ being even.

Without loss of generality, consider a URA consisting of $M=M_{x}\times M_{y}$ sensors with $M_{x}$ and $M_{y}$ being even. The method developed in this section can be easily extended to the cases when $M_{x}$ and $M_{y}$ have different parities. The geometry of the URA is shown in Figure 2, where the same icon means that those sensors have the same pattern and the same weighting coefficient.

The sensors of the URA are numbered in a consecutive way, starting with the bottom-left (m=1) and proceeding in the *x*-direction and then in the *y*-direction. Let $I_{m_{y}}^{k}$ be the indices of the sensors in the $m_{y}$th row in the [k+2sign(2.5−k)]th quadrant, i.e.,

(8a) $$\begin{array}{cc} I_{m_{y}}^{1} & =\left(m_{y}-1\right)M_{x}+\left\{1,\cdots ,\frac{M_{x}}{2}\right\}, \end{array}$$

(8b) $$\begin{array}{cc} I_{m_{y}}^{2} & =m_{y}M_{x}-\left\{0,\cdots ,\frac{M_{x}}{2}-1\right\}, \end{array}$$

(8c) $$\begin{array}{cc} I_{m_{y}}^{3} & =\left(M_{y}-m_{y}\right)M_{x}+\left\{1,\cdots ,\frac{M_{x}}{2}\right\}, \end{array}$$

(8d) $$\begin{array}{cc} I_{m_{y}}^{4} & =\left(M_{y}-m_{y}+1\right)M_{x}-\left\{0,\cdots ,\frac{M_{x}}{2}-1\right\}, \end{array}$$

where $m_{y}=1,\cdots ,M_{y}/2$. For example, if $M_{x}=6$ and $M_{y}=4$, then

(9a) $$\begin{array}{cccc} I_{1}^{1} & =\{1,2,3\}, & I_{2}^{1} & =\{7,8,9\}, \end{array}$$

(9b) $$\begin{array}{cccc} I_{1}^{2} & =\{6,5,4\}, & I_{2}^{2} & =\{12,11,10\}, \end{array}$$

(9c) $$\begin{array}{cccc} I_{1}^{3} & =\{19,20,21\}, & I_{2}^{3} & =\{13,14,15\}, \end{array}$$

(9d) $$\begin{array}{cccc} I_{1}^{4} & =\{24,23,22\}, & I_{2}^{4} & =\{18,17,16\}. \end{array}$$

Furthermore, let

(10) $$I^{k}=I_{1}^{k}\cup I_{2}^{k}\cdots \cup I_{M_{y}/2}^{k},\;k=1,2,3,4,$$

i.e., each $I^{k}$ contains the indices of sensors in one quadrant. By symmetry, we have

(11) $$w\left(I^{1}\right)=w\left(I^{2}\right)=w\left(I^{3}\right)=w\left(I^{4}\right).$$

Consequently,

(12) $$v_{n}^{H}w=\sum_{k=1}^{4}{\left[v_{n}\left(I^{k}\right)\right]}^{H}w\left(I^{k}\right)=u_{n}^{H}w\left(I^{1}\right),$$

where

(13) $$u_{n}=v_{n}\left(I^{1}\right)+v_{n}\left(I^{2}\right)+v_{n}\left(I^{3}\right)+v_{n}\left(I^{4}\right).$$

Equation (12) indicates that the dimension of the optimization variable (i.e., *g* and w) in Problem (7) can be reduced from M+1 to M/4+1.

In addition, due to the symmetry of array geometry, the array pattern also has symmetry in the ϕ-direction. For example, when specifying the sidelobe region, we only need to impose the constraints for $\phi \in [0,90^{\circ }]$ rather than $\phi \in [0,360^{\circ }]$. Strictly speaking, this simplification requires that the sensor patterns are also symmetric about the *x* and *y* axes, which is satisfied in practice because the sensor patterns in a large array usually depend weakly on ϕ. For example, the microstrip patch antenna and pyramidal/conical horn antenna are widely used in antenna arrays [46] (ch. 13), whose patterns are commonly modeled as functions that do not depend on ϕ [47] (ch. 6).

Now we can reformulate (7) as an SOCP problem whose primal and dual dimensions are $D_{p}=M/2+3N/4+1$ and $D_{d}=M/4+1$, respectively. Let

(14a) $$\begin{array}{cccc} y & =\left[\begin{array}{c} g\\ w(I^{1}) \end{array}\right], & b & =\left[\begin{array}{c} 1\\ 0 \end{array}\right], \end{array}$$

(14b) a0=0Re(u0),an=10,

(14c) An=00Re(un)Im(un),dm=0em

for n=1,⋯,N/4 and m=1,⋯,M/4, where $e_{m}$ is the *m*th column of the identity matrix of order M/4. Then Problem (7) can be reformulated as

(15a) $$\begin{array}{cc} \min_{y\in R^{(M/4)+1}}\; & b^{T}y, \end{array}$$

(15b) $$\begin{array}{cc} \; & 0\leq a_{0}^{T}y-1, \end{array}$$

(15c) $$\begin{array}{cc} \; & 0\leq d_{m}^{T}y-\alpha_{m}, \end{array}$$

(15d) $$\begin{array}{cc} \; & 0\leq -d_{m}^{T}y+\beta_{m}, \end{array}$$

(15e) $$\begin{array}{cc} \; & ∥A_{n}^{T}y∥\leq a_{n}^{T}y, \end{array}$$

which is a standard (dual) SOCP problem [43]. Note that the constraint $a_{0}^{T}y=1$ has been replaced by $0\leq a_{0}^{T}y-1$ in order to express (15) in the standard form. This replacement does not change the solution of (15) because $w(I^{1})$ should become as small as possible to minimize the PSLL. Therefore, the inequality in (15b) will become an equality at the optimal point.

### 3.2. Fast Amplitude Weighting for UHA Using Rotational Symmetry

**Figure 3:**
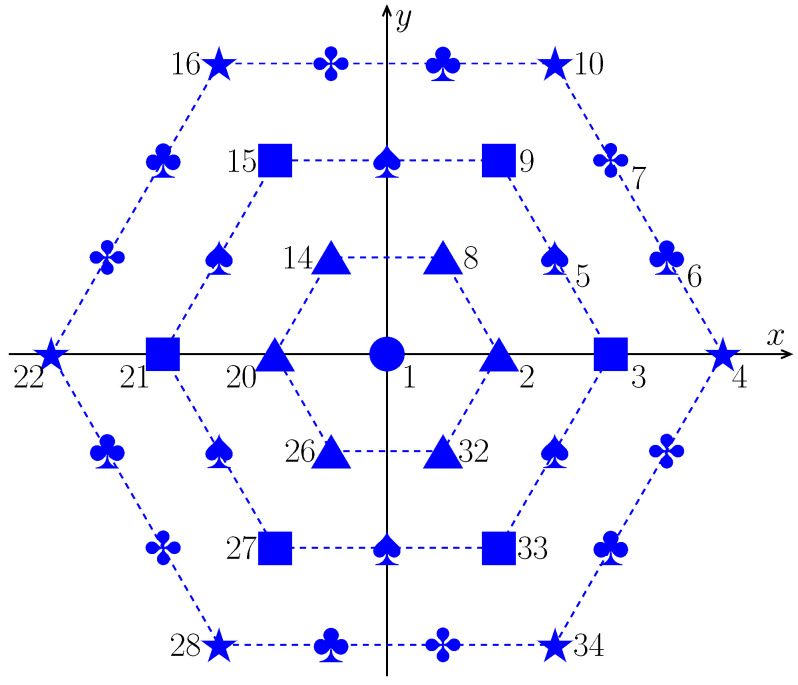
The symmetry of a UHA with 37 sensors.

The geometry of the UHA is shown in Figure 3, where the same icon means that those sensors have the same pattern and the same weighting coefficient. It can be seen that the UHA is rotationally symmetric about the origin.

Suppose the UHA consists of *C* hexagons (dashed lines in Figure 3); then, there are

(16) M=1+6+12+⋯+6C=3C(C+1)+1

sensors in the array. In the region of $0^{\circ }\leq \phi <60^{\circ }$, there are (including the origin)

(17) $$\frac{M-1}{6}+1=\frac{C(C+1)}{2}+1=H+1$$

sensors, where

(18) $$H=\frac{M-1}{6}\;\mathrm{and}\;M=6H+1\;.$$

For example, in Figure 3, C=3, M=37, and H=6.

The numbering system of the UHA is as follows:

- The H+1 sensors in the region of $0^{\circ }\leq \phi <60^{\circ }$ are numbered in a consecutive way, starting with the origin (m=1) and proceeding in the *x*-direction and then in the *y*-direction (m=2,⋯,H+1).
- If the position of a sensor is obtained by rotating the *m*th sensor (m=2,⋯,H+1) with an angle of $k\times 60^{\circ }$ (k=1,⋯,5), then this sensor is numbered by m=2+kH.

By using symmetry, we only have to optimize the weighting coefficients in the region of $0^{\circ }\leq \phi <60^{\circ }$. Let

(19) $$I^{k}=\left\{kH+2,kH+3,\cdots ,kH+\left(H+1\right)\right\}$$

for k=0,1,⋯,5, then

(20) $$w\left(I^{0}\right)=w\left(I^{1}\right)=\cdots =w\left(I^{5}\right).$$

Consequently,

(21) $$\begin{array}{cc} v_{n}^{H}w & =v_{n1}^{*}w_{1}+\sum_{k=0}^{5}{\left[v_{n}\left(I^{k}\right)\right]}^{H}w\left(I^{k}\right)\\ \; & =v_{n1}^{*}w_{1}+u_{n}^{H}w\left(I^{1}\right), \end{array}$$

where

(22) $$u_{n}=v_{n}\left(I^{0}\right)+v_{n}\left(I^{1}\right)+\cdots +v_{n}\left(I^{5}\right).$$

Equation (21) indicates that the dimension of w in Problem (7) can be reduced from M+1 to (M−1)/6+2.

Similar to the case of URA, we only need to impose the constraints for $\phi \in [0,90^{\circ }]$ rather than $\phi \in [0,360^{\circ }]$. And (7) can be reformulated as (15) with

(23a) $$\begin{array}{cccc} y & =\left[\begin{array}{c} g\\ w_{1}\\ w(I^{1}) \end{array}\right], & b & =\left[\begin{array}{c} 1\\ 0 \end{array}\right], \end{array}$$

(23b) a0=0Re(v01)Re(u0),an=10,

(23c) An=00Re(vn1)Im(vn1)Re(un)Im(un),dm=0em

for n=1,⋯,N/4 and m=1,⋯,H/6+1, where $e_{m}$ is the *m*th column of the identity matrix of order H+1. The primal and dual dimensions of this simplified problem are $D_{p}=2H+3N/4+2$ and $D_{d}=H+1$, respectively.

## 4. Numerical Results and Analyses

### 4.1. Experiment 1: URA with Mirror Symmetry

A URA consisting of 16×16 isotropic sensors is used in the first experiment. Because the array pattern is symmetric about the xy-plane, only the upper half-space is shown when plotting the array pattern. Problems (7) and (15) can be solved by the CVX package. However, to provide a user-friendly interface, CVX may introduce auxiliary variables when transforming a given problem to a standard one. These redundant variables may reduce the computational efficiency of the solving processes. Therefore, we use the algorithm described in [31,43] to solve Problems (7) and (15), whose solutions are denoted by $w_{\left(7\right)}$ and $w_{\left(15\right)}$, respectively.

**Figure 4:**
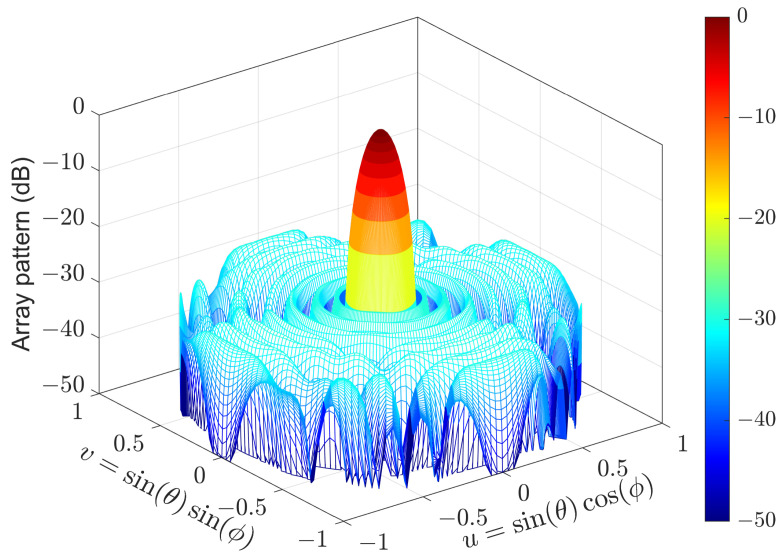
Array pattern of the 16×16 URA synthesized by (15).

**Figure 5:**
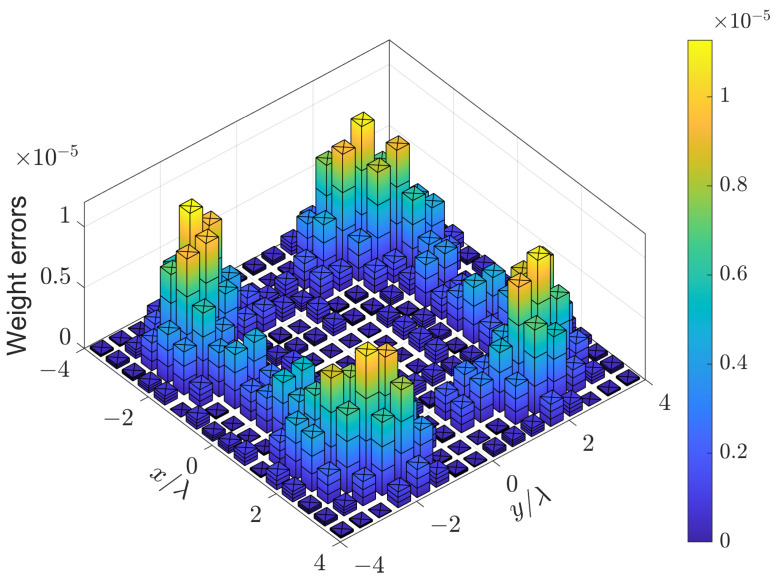
Weighting errors of the 16×16 URA synthesized by (15) and (7).

The region of sidelobes is ${\left[10,90\right]}^{\circ }\times {\left[0,360\right]}^{\circ }$, which is sampled by $\delta_{\theta }=2^{\circ }$ and $\delta_{\phi }=4^{\circ }$. To prevent negative weighting coefficients, $\alpha_{m}$ are usually set to 0. Because $w_{m}=1/M$ for uniform weighting, $\beta_{m}$ are usually set to μ/M, where μ is near 2. In this example, $\beta_{m}=2.1/M$. The pattern synthesized by (15) is shown in Figure 4, which is the same as that synthesized by (7)). The weighting errors between $w_{\left(15\right)}$ and $w_{\left(7\right)}$ are shown in Figure 5. It can be seen that the difference between $w_{\left(15\right)}$ and $w_{\left(7\right)}$ is negligible. The relative error is

(24) $$\frac{∥w_{\left(15\right)}-w_{\left(7\right)}∥}{∥w_{\left(7\right)}∥}=0.07\%\;,$$

which means that Problem (7) also gives symmetric weighting coefficients.

The CPU time $T_{\mathrm{cpu}}$ of solving (7) and (15) on a computer with four 2.4 GHz CPUs as well as the PSLLs of the synthesized patterns are listed in Table 2. It can be seen that (7) and (15) produce the same PSLL. However, solving (15) is 12 times faster than solving (7). The results when (7) and (15) are solved by CVX [29] are also provided, which indicates that solving (15) is 28 times faster than solving (7). In addition, the CVX package spends more CPU time than the solver based on [31,43]. The dimensions of the primal and dual variables involved in (7) and (15) are also listed in Table 2, showing that the dimension of the optimization problem is reduced by 75% using the mirror symmetry of the URA.

**Figure 6:**
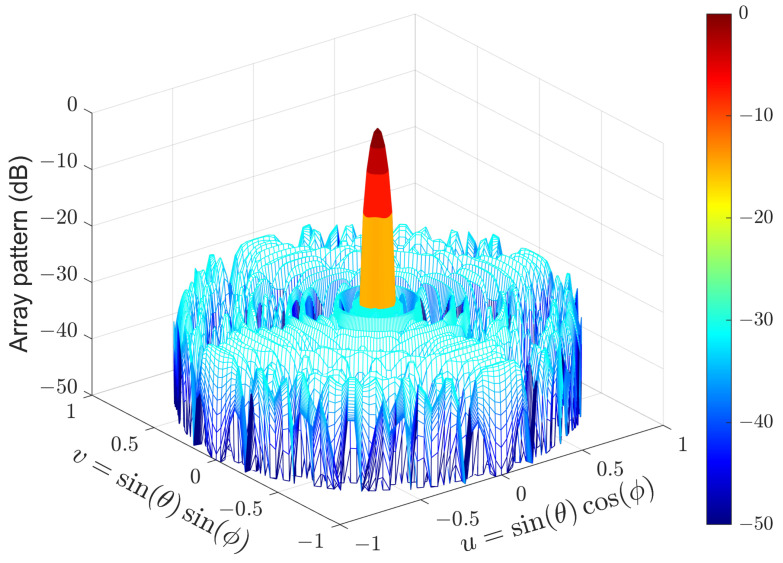
Array pattern of the 32×32 URA synthesized by (15).

Another URA consisting of 32×32 isotropic sensors is used in the second experiment. The region of sidelobes is ${\left[5,90\right]}^{\circ }\times {\left[0,360\right]}^{\circ }$, which is sampled by $\delta_{\theta }=1^{\circ }$ and $\delta_{\phi }=2^{\circ }$. The parameters $\alpha_{m}$ and $\beta_{m}$ are set to 0 and 1.9/M, respectively. The pattern synthesized by (15) is shown in Figure 6, while the CPU time and PSLL are listed in Table 2. It can be seen that (15) produces the same PSLL as that of (7). However, solving (15) is 158 times faster than solving (7). On the other hand, solving (15) is 83 times faster than solving (7) by using the CVX [29] toolbox.

**Figure 7:**
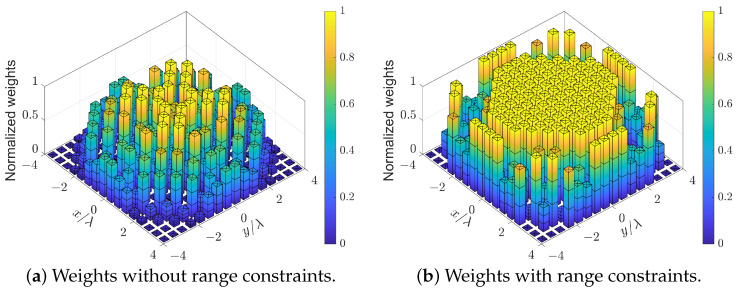

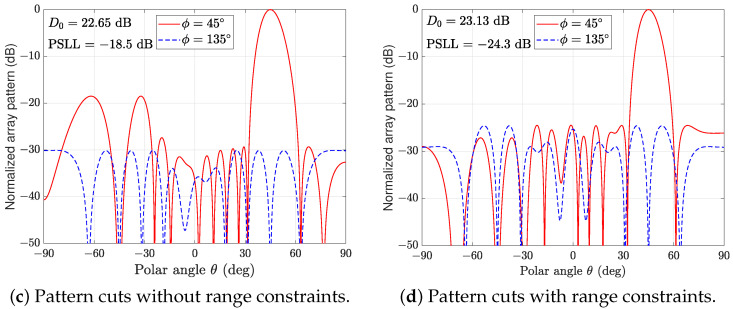
The effect of dynamic range constraints when the array is steered to (45°, 45°).

The effect of dynamic range constraints $\alpha_{m}\leq w_{m}\leq \beta_{m}$ is shown in Figure 7, where the synthesized patterns of the 16×16 URA with and without the dynamic range constraints are compared. The lower and upper bounds of the weighting coefficients are $\alpha_{m}=0$ and $\beta_{m}=1.5/M$ respectively. It can be seen that the weighting coefficients have a smaller fluctuation when the dynamic range constraints are imposed, which can enhance the robustness of array patterns [48]. For example, when the array is steered to $\left(45^{\circ },45^{\circ }\right)$, the pattern with dynamic range constraints has a higher directivity (improved by 0.5 dB) and a lower PSLL (improved by 5.8 dB) than the pattern without dynamic range constraints.

### 4.2. Experiment 2: UHA with Rotational Symmetry

**Figure 8:**
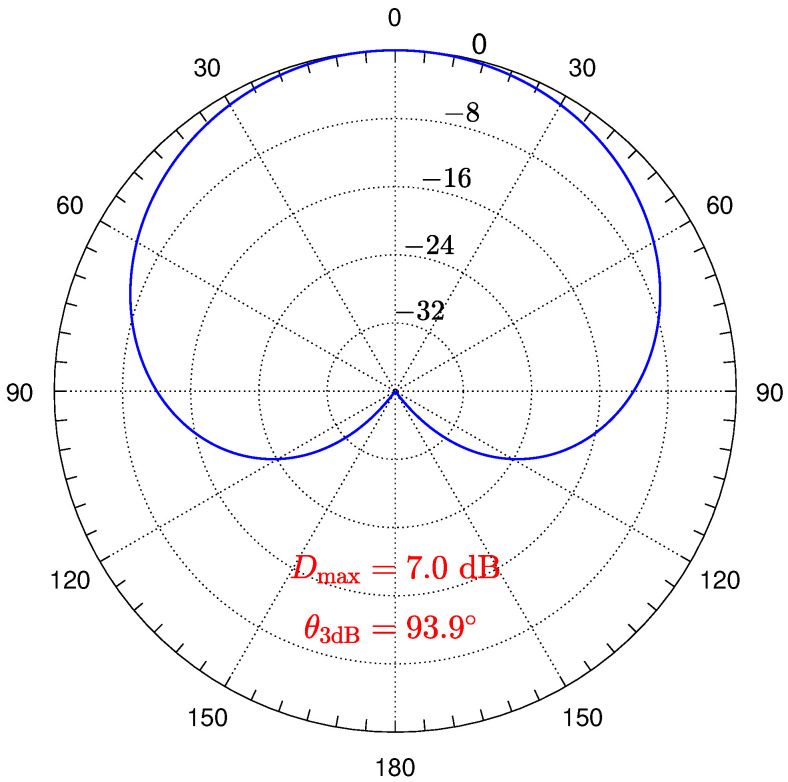
The pattern of $\cos^{4}\left(\theta /2\right)$ with directivity $D_{\max}=7.0$ dB and 3 dB beamwidth $\theta_{3\mathrm{dB}}=93.9^{\circ }$.

A UHA consisting of 10 hexagons (331 sensors) is used in the third experiment. All sensors have the same pattern, given by

(25) $$f_{m}\left(\theta ,\phi \right)={\left[\frac{1+\cos(\theta )}{2}\right]}^{2}=\cos^{4}\left(\theta /2\right)\;,$$

which is shown in Figure 8. Because this pattern is not symmetric about the xy-plane, the full space is shown when plotting the array patterns.

**Figure 9:**
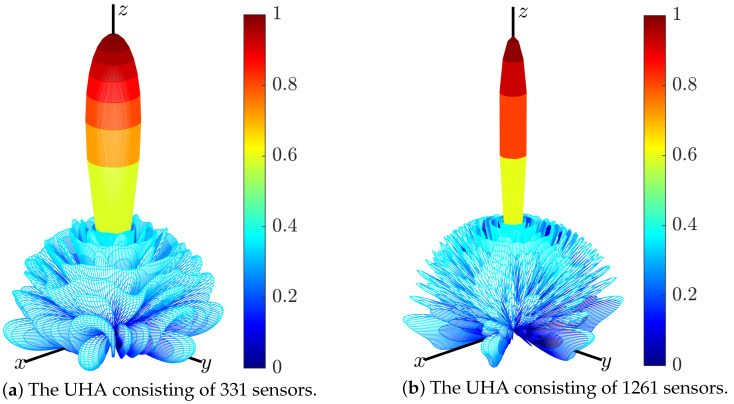
Array patterns of the UHA synthesized by (15).

**Figure 10:**
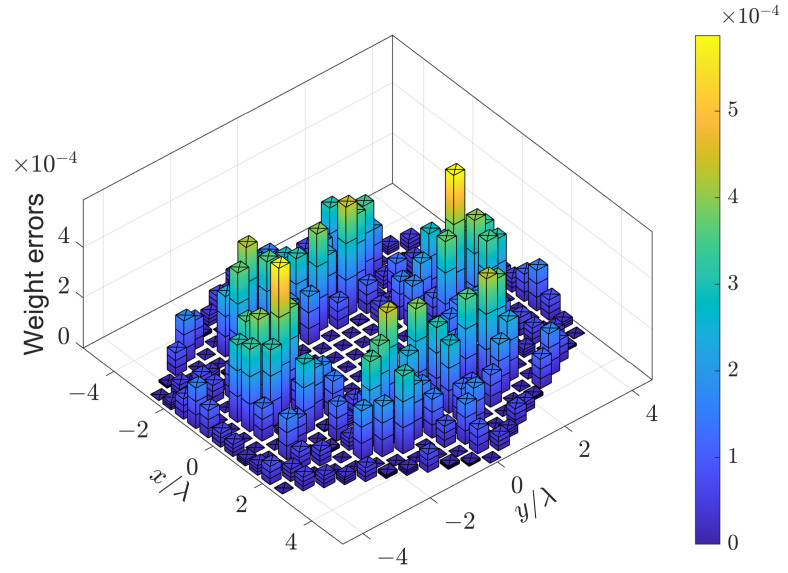
Weighting errors of the UHA with 10 hexagons synthesized by (15) and (7).

The region of sidelobes is ${\left[9,180\right]}^{\circ }\times {\left[0,360\right]}^{\circ }$, which is sampled by $\delta_{\theta }=2^{\circ }$ and $\delta_{\phi }=4^{\circ }$. The parameters $\alpha_{m}$ and $\beta_{m}$ are set to 0 and 1.8/M, respectively. The pattern synthesized by (15) is shown in Figure 9a, which is the same as that synthesized by (7)). The weighting errors between $w_{\left(15\right)}$ and $w_{\left(7\right)}$ are shown in Figure 10. It can be seen that the difference between $w_{\left(15\right)}$ and $w_{\left(7\right)}$ is negligible. The relative error is

(26) $$\frac{∥w_{\left(15\right)}-w_{\left(7\right)}∥}{∥w_{\left(7\right)}∥}=0.02\%\;,$$

which means that Problem (7) also gives symmetric weighting coefficients. The CPU time $T_{\mathrm{cpu}}$ of solving (7) and (15) as well as the PSLL are listed in Table 3. It can be seen that (7) and (15) produce the same PSLL. However, solving (15) is 19 times faster than solving (7). The results when (7) and (15) are solved by CVX [29] are also provided, which indicates that solving (15) is 49 times faster than solving (7). The dimensions of the primal and dual variables involved in (7) and (15) are also listed in Table 3. The results indicate that the number of constraints rather than the number of sensors dominates the dimension of the optimization problem.

Another UHA consisting of 20 hexagons (1261 sensors) is used in the fourth experiment. The region of sidelobes is ${\left[5,90\right]}^{\circ }\times {\left[0,360\right]}^{\circ }$, which is sampled by $\delta_{\theta }=1^{\circ }$ and $\delta_{\phi }=2^{\circ }$. The parameters $\alpha_{m}$ and $\beta_{m}$ are set to 0 and 1.7/M, respectively. The pattern synthesized by (15) is shown in Figure 9b, while the CPU time and PSLL are listed in Table 3. It can be seen that (15) produces the same PSLL as that of (7). However, solving (15) is 284 times faster than solving (7). On the other hand, solving (15) is 133 times faster than solving (7) by using the CVX [29] toolbox.

## 5. Conclusions and Discussion

A fast low-sidelobe pattern synthesis method was studied in this paper, which utilizes the symmetry of array geometry to reduce the dimension of the optimization variables as well as the number of constraints. Different from the analytical approaches, the proposed method was modeled by an SOCP problem which can take into account the sensor pattern and the dynamic range of the weighting coefficients. Simulation experiments showed that, for a URA with 1024 sensors, the computational efficiency was improved by a factor of 158, while for a UHA with 1261 sensors, the improvement factor was 284. In addition, the proposed method can be easily extended to other symmetric arrays.

The improvement of computational efficiency in the second experiment is larger than that in the first experiment. This is because the primal and dual dimensions of (7) in the first experiment are $D_{p}=$ 11,706 and $D_{d}=257$, while in the second experiment, they are $D_{p}=$ 48,747 and $D_{d}=1025$. Therefore, constructing the $D_{p}\times D_{p}$ matrix in the interior-point method dominates the computations in the first experiment, while solving the $D_{p}\times D_{p}$ linear systems of equations dominates the computations in the second experiment. For the same reason, the improvement of computational efficiency in the fourth experiment is larger than that in the third experiment.

It is worth remarking that there may be different types of symmetry in one array (e.g., the mirror symmetry and rotational symmetry in a square array). In such cases, either symmetry can be exploited depending on the array pattern to be synthesized. Finally, Table 2 and Table 3 show that the direct implementation of the SOCP solver described in [31,43] is faster than the CVX toolbox [29]. By checking the interior parameters, it is found that the dimension of the optimization problem in CVX is larger than that of the standard form. Therefore, redundant variables are introduced in CVX when solving a convex problem that is not in its standard form. Another possible reason is that before solving the given problem, pre-examination is performed by the CVX toolbox to select a proper algorithm, which takes extra time.

Applications of array symmetries discussed in this paper are not limited to amplitude weighting; they can also be utilized in other array pattern synthesis problems such as sparse array optimization. Apart from convex optimization, other numerical optimization-based methods can also leverage this geometry property. By exploiting array symmetries, advantages arise not only on the improvement of computational efficiency; from an engineering point of view, symmetry also eases the difficulty in manufacture of the sensor arrays. With the development of extremely large-scale sensor arrays, we believe that the applications of array symmetries will attract more and more attention. In addition, future works could focus on their extension to semi-symmetric arrays, where partial symmetry exists in the array geometry.

## Figures and Tables

**Table 1:** Variable definitions for Problem (7).

Variable/Parameter	Meaning
*g*	the maximum array gain in sidelobe region
$v_{0}$	steering vector of the scan angle
w	weighting coefficients (optimization variables)
$v_{n}$	steering vectors in sidelobe region
$\alpha_{m}$	lower bounds of the weighting coefficients
$\beta_{m}$	upper bounds of the weighting coefficients

**Table 2:** The PSLL and CPU time for URA.

Problem	Solver	$M_{x}\times M_{y}$	$D_{p}$	$D_{d}$	PSLL (dB)	$T_{\mathrm{cpu}}$ (s)
(7)	[31] + [43]	16×16	11,706	257	−29.6	15.3
(7)	CVX [29]	16×16	/	/	−29.6	61.5
(15)	[31] + [43]	16×16	2958	65	−29.6	1.3
(15)	CVX [29]	16×16	/	/	−29.6	2.2
(7)	[31] + [43]	32×32	48,747	1025	−30.3	2884.6
(7)	CVX [29]	32×32	/	/	−30.3	4088.1
(15)	[31] + [43]	32×32	12,381	257	−30.3	18.3
(15)	CVX [29]	32×32	/	/	−30.3	49.1

**Table 3:** The PSLL and CPU time for UHA.

Problem	Solver	*M*	$D_{p}$	$D_{d}$	PSLL (dB)	$T_{\mathrm{cpu}}$ (s)
(7)	[31] + [43]	331	11,856	332	−30.9	20.7
(7)	CVX [29]	331	/	/	−30.9	92.3
(15)	[31] + [43]	331	2942	57	−30.9	1.1
(15)	CVX [29]	331	/	/	−30.9	1.9
(7)	[31] + [43]	1261	49,221	1262	−31.2	3859.0
(7)	CVX [29]	1261	/	/	−31.2	5480.6
(15)	[31] + [43]	1261	12,291	212	−31.2	12.8
(15)	CVX [29]	1261	/	/	−31.2	41.1

## Data Availability

Data are contained within the article.
